# Selection of lansoprazole from an FDA-approved drug library to inhibit the Alzheimer’s disease seed-dependent formation of tau aggregates

**DOI:** 10.3389/fnagi.2024.1368291

**Published:** 2024-03-26

**Authors:** Ahmed Imtiaz, Shotaro Shimonaka, Mohammad Nasir Uddin, Montasir Elahi, Koichi Ishiguro, Masato Hasegawa, Nobutaka Hattori, Yumiko Motoi

**Affiliations:** ^1^Department of Diagnosis, Prevention and Treatment of Dementia, Juntendo University Graduate School of Medicine, Bunkyo-ku, Tokyo, Japan; ^2^Department of Neurology, Juntendo University School of Medicine, Hongo, Bunkyo-ku, Tokyo, Japan; ^3^Research Institute for Diseases of Old Age, Juntendo University Graduate School of Medicine, Bunkyo-ku, Tokyo, Japan; ^4^Department of Biochemistry & Molecular Biology, Faculty of Life Science, Mawlana Bhashani Science & Technology University, Tangail, Bangladesh; ^5^Center for Birth Defect Research, University of Maryland School of Medicine, Baltimore, MD, United States; ^6^Department of Brain and Neuroscience, Tokyo Metropolitan Institute of Medical Science, Setagaya-ku, Tokyo, Japan; ^7^Medical Center for Dementia, Juntendo University Hospital, Bunkyo-ku, Tokyo, Japan

**Keywords:** tau, aggregation, Alzheimer’s disease, filament, seed, drug reposition, FDA-approved drug

## Abstract

The efficacy of current treatments is still insufficient for Alzheimer’s disease (AD), the most common cause of Dementia. Out of the two pathological hallmarks of AD amyloid-β plaques and neurofibrillary tangles, comprising of tau protein, tau pathology strongly correlates with the symptoms of AD. Previously, screening for inhibitors of tau aggregation that target recombinant tau aggregates have been attempted. Since a recent cryo-EM analysis revealed distinct differences in the folding patterns of heparin-induced recombinant tau filaments and AD tau filaments, this study focused on AD seed-dependent tau aggregation in drug repositioning for AD. We screened 763 compounds from an FDA-approved drug library using an AD seed-induced tau aggregation in SH-SY5Y cell-based assay. In the first screening, 180 compounds were selected, 72 of which were excluded based on the results of lactate dehydrogenase assay. In the third screening with evaluations of soluble and insoluble tau, 38 compounds were selected. In the fourth screening with 3 different AD seeds, 4 compounds, lansoprazole, calcipotriene, desogestrel, and pentamidine isethionate, were selected. After AD seed-induced real-time quaking-induced conversion, lansoprazole was selected as the most suitable drug for repositioning. The intranasal administration of lansoprazole for 4 months to AD seed-injected mice improved locomotor activity and reduced both the amount of insoluble tau and the extent of phosphorylated tau-positive areas. Alanine replacement of the predicted binding site to an AD filament indicated the involvement of Q351, H362, and K369 in lansoprazole and C-shaped tau filaments. These results suggest the potential of lansoprazole as a candidate for drug repositioning to an inhibitor of tau aggregate formation in AD.

## 1 Introduction

The number of people living with dementia was estimated to be 55 million in 2019 and is expected to increase to 139 million by 2050 (International, ASD, 2022). With an aging population, the economic burden and caregiver stress for dementia will increase. Alzheimer’s disease (AD) is the most common form of dementia and its pathological hallmarks are senile plaques primarily composed of amyloid-β and intraneuronal neurofibrillary tangles, the main component of which is the tau protein. Many clinical trials on disease-modifying therapies targeting amyloid-β have been unsuccessful. Lecanemab, a humanized IgG1 monoclonal antibody against amyloid-β, has been approved by the U.S. Food and Drug Administration and the Japanese Ministry of Health, Labor and Welfare; however, the results from clinical trials showed that Lecanemab slowed cognitive decline rather than improve cognition or halt decline, and there were risks such as brain edema and microhemorrhage (Van Dyck et al., 2023). Since tau pathology correlates with the cognitive status in AD (Wilcock and Esiri, 1982; Braak et al., 2006), tau-targeting strategies are attracting increasing interest.

Under physiological conditions, tau is a highly soluble and natively unfolded protein; however, under pathological conditions, the aggregation of tau into amyloid fibrils characterizes a series of neurodegenerative diseases designated as tauopathies, including AD (Goedert et al., 2017). Tau pathology appears to spread through intercellular propagation, requiring prion-like spreads, showing its conversion from a soluble monomeric state to self-propagating aggregates rich in a β-sheet structure. These tau assemblies stably maintain unique conformations *in vivo* that seed the native monomer to amyloid aggregates. In mice, an injection of tau inclusions induced nerve cells to form intracellular inclusions at the injection site, from which they spread to distant brain regions (Clavaguera et al., 2009; Guo et al., 2016). In our laboratory, an injection of brain extracts from human AD into the unilateral mouse hippocampus resulted in silver-positive tau pathology in the fimbria, locus coeruleus, and contralateral hippocampus that was accompanied with learning deficits (Hayashi et al., 2021). At the cellular level, as seeds, pathological tau conformers serve as templates to recruit the native protein into growing assemblies (Vaquer-Alicea et al., 2021). The cryo-EM structures of tau fibrils isolated from AD brains revealed that filament cores comprise two C-shaped identical protofilaments comprising residues 306–378 of the tau protein, which adopt a cross-β/β-helix structure and define seeds for aggregation (Fitzpatrick et al., 2017). On the other hand, the structure of heparin-induced tau filaments is polymorphic and different from that in AD (Zhang et al., 2019). 2N4R tau assembles into multiple types of filaments, and the structures of three types have similar “kinked hairpin” folds. The unique conformation of each tauopathy may affect the efficacy of small molecules in therapy (Vaquer-Alicea et al., 2021).

The majority of high-content screening assays (HCS) for tau aggregation inhibitors have used recombinant tau and the compounds identified in each screening varies. High throughput screening (~51,000 compounds) was conducted with the bacterially expressed recombinant tau fragment K18 comprising four repeats in the microtubule-binding domain in the presence of heparin, and two 2,3-di(furan-2-yl)-quinoxalines were confirmed to be inhibitors of tau fibrillization (Crowe et al., 2007). A heparin-induced aggregation assay combined with a combination of conventional docking and machine learning-based screening selected two compounds from a pilot set of 1,000 compounds (Baggett and Nath, 2018). In cell-based HCS to examine the effects of 1,649 compounds on the inhibition of tau aggregation in N2a cells expressing the pro-aggregant repeat domain of the tau protein (tau ^RDΔK^), inhibitors of protein kinases were found to suppress tau aggregation, while inhibitors of deacetylases enhanced aggregation (Pickhardt et al., 2019). Hyperphosphorylated recombinant tau was used in the *in vitro* screening of a 1,280-FDA approved library, and R-(−)apomorphine and raloxifene were identified as phosphorylated tau aggregation inhibitors (Liu et al., 2020). A primary rat cortical neuron assay seeded with AD brain tissues for 14 days before the assessment of tau inclusions through immunostaining and imaging was applied to the screening of the Prestwick library, and a large number of inhibitors were identified, including four dopamine D2 receptor antagonists (Crowe et al., 2020). HEK293T cell lines stably expressing tau-K18 P301S-eYFP were incubated with AD brain tissues and this model was used for FDA-approved small molecule drugs (~1700 compounds) and the ChemBridge CNS-set, a library of approximately 60,000 compounds, and the drugs identified are confidential (Seidler et al., 2022).

Based on the distinct folds of AD filaments, recombinant tau, and other tauopathies, the present study focused on AD seed-templated tau both in a cellular environment and under cell-free conditions (AD seed-induced real-time quaking-induced conversion: RT-QuIC) with the aim of repurposing FDA-approved drugs for the inhibition of AD tau aggregation. We screened an FDA-approved drug library comprising 763 compounds using AD seed-dependent SH-SY5Y cells to identify compounds that effectively inhibit seed-dependent tau aggregation. In the first screening, AD brain cell line models were treated with 763 compounds from the library. We then performed a cytotoxicity LDH assay, an assessment of soluble and insoluble tau, a triplication analysis using multiple AD cases, and an *in vitro* AD seed-dependent RT-QuIC assay. We identified the proton pump inhibitor (PPI), lansoprazole, as the most suitable drug for repositioning. We then examined the effects of lansoprazole on tau aggregation using an AD seed-injected mouse model.

## 2 Materials and methods

### 2.1 Preparation of human AD brain seeds

Human brain samples were acquired from the brain bank of Juntendo University Hospital. Ethical approval for the use of human brain samples was obtained from the Ethics Committee of Juntendo University School of Medicine (approval number: 2012068). Frontal cortices from patients with pathologically confirmed AD (*n* = 3) were utilized for seed preparation (refer to Supplementary Table S1 for details). Frozen brain tissues were sectioned into 0.2-to 0.3-g blocks and promptly homogenized in 2 mL of A68 buffer (10 mM Tris–HCl pH 7.5, 10% sucrose, 0.8 M NaCl, and 1 mM EGTA) using a Dounce homogenizer. Lysates were sonicated with an ultrasonic homogenizer (VPe050 N, TAITEC Corporation, Japan) for 1 min (power 25.0%) and centrifuged at 3,000 × *g* at 4°C for 10 min. The resulting supernatants were stored at −80°C and the pellets were discarded [10].

### 2.2 Cell culture, transfection, seed induction, and drug addition

SH-SY5Y cells were cultured in Dulbecco’s modified Eagle’s medium/F12 DMEM (Merck, Germany), supplemented with 10% fetal calf serum, MEM Non-Essential Amino Acid Solution (Gibco), and Penicillin–Streptomycin-Glutamine (Gibco). Cells were maintained at 37°C in a humidified atmosphere of 5% CO_2_. Cells were grown to 70–80% confluency in collagen-coated 24-well culture dishes (Merck) and used for transfection and seeding. The transfection of the expression plasmid [pcDNA3_Tau-CTF24(242–441)] (Matsumoto et al., 2015) was performed using X-tremeGENE9 (Roche, Switzerland). Tau seeds (patient brain lysates) were suspended in Opti-MEM (Thermo Fisher, United States), mixed with Multifectam (Promega, United States), incubated at room temperature for 30 min, and introduced into cells. A total of 763 drugs from the Screen-Well FDA approved Drug Library V2 were added at a concentration of 10 μM and incubated for 48 h. Dimethyl sulfoxide (DMSO) was added at a concentration of 10 μM as the control.

### 2.3 Lactate dehydrogenase (LDH) assay

The LDH Cytotoxicity Detection Kit from Takara Bio (Cat# MK401) was employed for the LDH assay and performed according to the manufacturer’s protocol. To define 100% LDH activity, 100% Triton-X was added to each well (final 2%), while 0.5 μL of DMSO was added to each well instead of drugs to define 0% LDH activity. SH-SY5Y cells were transfected with pcDNA3_Tau-CTF24, and AD seeds and drugs were added. Each of the 180 drugs selected in the first screening was tested twice. Each drug was repeated twice for 180 drugs. After a 48-h incubation, media were collected for the LDH assay. Media collected 10 min after the addition of Triton-X were considered to exhibit 100% LDH activity. Media from DMSO-treated wells were considered to have 0% LDH activity.

### 2.4 Purification of recombinant tau

*Escherichia coli* BL21 (DE3) cells were transformed with the expression plasmid pRK172 harboring dGAE tau sequences (297–391) (Al-Hilaly et al., 2020). After the mass culture of transformed BL21 cells in 500 mL of 2× YT medium, recombinant tau proteins were purified as previously described (Shimonaka et al., 2020). After dialysis against 30 mM Tris–HCL pH 7.5 overnight, samples were centrifuged at 10,000 × *g* for 20 min and the supernatant was used. The concentration of the tau protein was estimated using a BCA Protein Assay Kit (Pierce).

### 2.5 Seed-induced RT-QuIC

Healthy brain lysates as the control were prepared using the same method as that described for AD seeds. AD brain seeds and healthy brain lysates were diluted 1,000× in phosphate buffer pH 7.4 (PB). In a 96-well optical-bottom plates with a polymer base (Thermo Scientific, 265,301), 1 μL of the brain lysate dilution was added to 99 μL of the RT-QuIC reaction mixture containing 7.5 μM tau dGAE, 10 mM dithiothreitol, 10 μM Thioflavin T (ThT), and 0.05% Tween 20 in PB. Drugs were added at a concentration of 50 μM. Zirconia/Silica beads (0.5 mm, BioSpec Products, 11079105z) were then added. After the plate was covered with sealing tape (Greiner Bio-one; J676060), it was incubated at 30°C in the FLUOstar Omega (BMG LABTECH) plate reader. RT-QuIC was performed with double orbital shaking at 600 rpm (60 s on/60 s off) and ThT fluorescence (448 nm excitation/482 nm emission) was recorded for 192 cycles.

### 2.6 Sarkosyl-insoluble tau extraction

Approximately 0.25 g of AD brain tissue was homogenized using a Dounce homogenizer in 5 mL of A68 buffer with 2% Sarkosyl and incubated in a water bath at 37°C for 30 min. Lysates were sonicated using an ultrasonic homogenizer for 15 s (power 30.0%) and centrifuged at 20,000 × *g* at 25°C for 10 min. The resulting pellets were washed with sterile saline solution and centrifuged at 100,000 × *g* at 25°C for 5 min. Pellets were mixed in 25 μL 1× PBS (pH 7.4) using an ultrasonic homogenizer for 15 s (power 15.0%). The mixture was centrifuged at 10,000 × *g* at 25°C for 30 min. The resulting supernatant was used for a stereotactic injection and contained tau fibrils. In the immunoblot analysis, 2 μL of the supernatant was mixed with SDS-sample buffer and subjected to 15% polyacrylamide gel SDS-PAGE. Proteins in gels were transferred onto a polyvinylidene difluoride membrane (Millipore) and blocked with 3% gelatin. Membranes were washed with 1× PBS and incubated for 2 h with a biotin-labeled secondary antibody (Vector) at room temperature. Protein bands were visualized using an ABC staining kit (Vector) (refer to Supplementary Table S2).

### 2.7 Stereotaxic surgery

All experiments were performed in accordance with the Guidelines for Animal Experiments of Juntendo University (Permit number: 2023157). Mice aged between 5 and 7 months were anesthetized with 2.5% isoflurane inhalation. Using a 29-G Hamilton syringe, the caudate putamen (A/P, +0.4 mm from the bregma; L, +2.0 mm; D/V, −3.2 mm) was unilaterally infused with 2.5 μL sarkosyl-insoluble tau at a speed of 100 nL per minute. After the injection, the needle was kept in place for an additional 10 min before gentle withdrawal. The surgical area was cleaned with sterile saline, and the incision was sutured using medical adhesive. Mice were monitored until recovery from anesthesia and checked daily after surgery. Sarkosyl-insoluble tau was injected into 25 C57BL/6 J mice.

### 2.8 Intranasal lansoprazole treatment in mice

One week after stereotactic surgery, mice were held in an upright to supine position, and 10 μL (5 μL in each naris) of the DMSO control (*n* = 12) or 10 mg/mL lansoprazole dissolved in DMSO (*n* = 13) was slowly injected 5 days a week for 16 weeks according to the intranasal rifampicin protocol (Umeda et al., 2022). Mice were held in the same position for 15–20 s before being returned to the cage. All treatments were performed without the prior habituation of mice to handling.

### 2.9 Behavioral test

**Figure 1:**
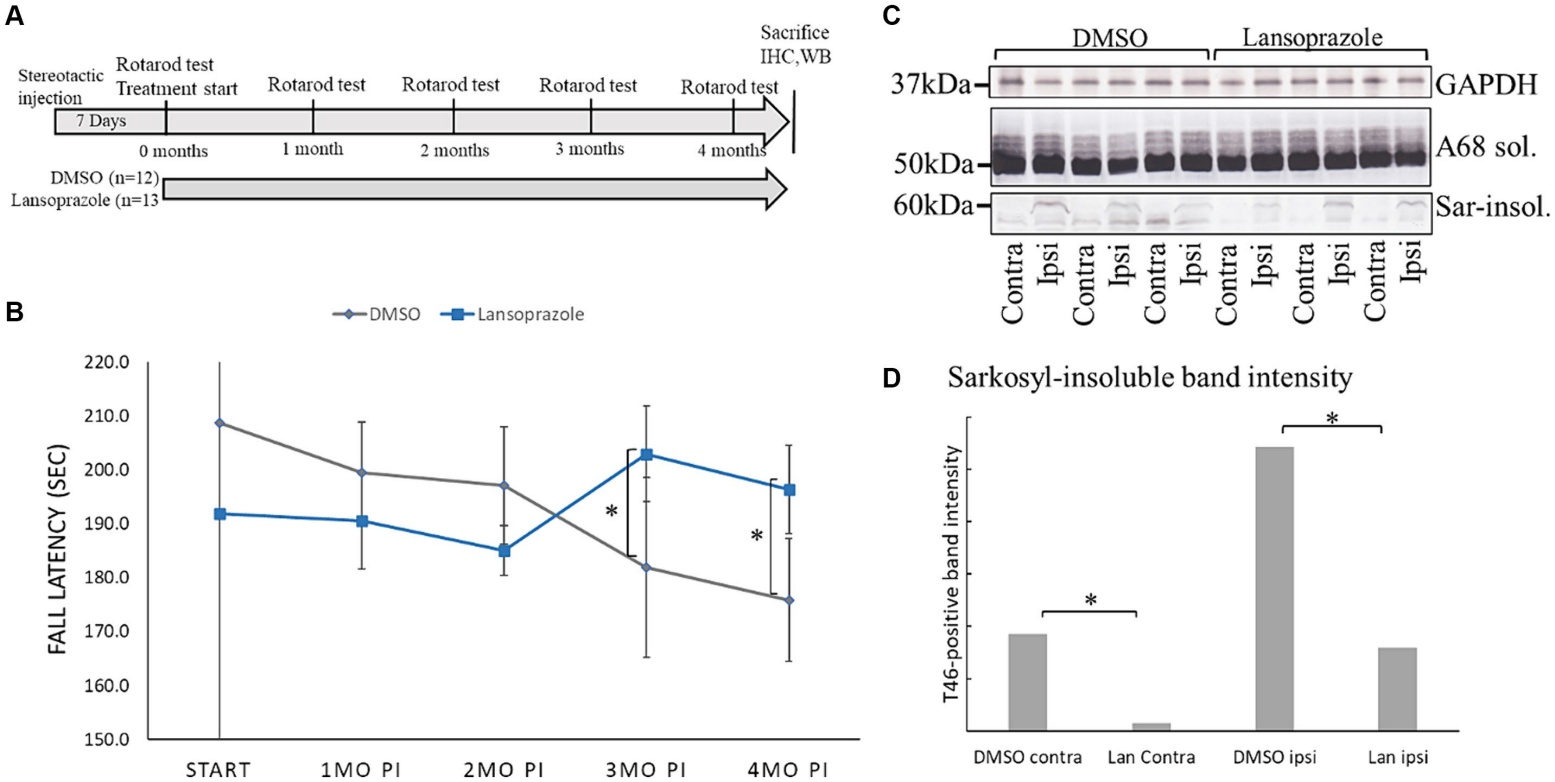
Lansoprazole improved locomotor activity and decreased insoluble tau in AD-seed injected mice. **(A)** Timeline of the AD seed-injected tau propagation mouse model. One week was allowed for the injection site to heal. The first rotarod treadmill test was performed before the initiation of intranasal drug administration. The rotarod test was conducted once every month until 4 months. **(B)** Lansoprazole effects on motor performance. At the beginning of the treatments, fall latency was shorter in the lansoprazole-treated group (n = 13) than in the DMSO-treated (n = 12) group. After 3 months of treatment, fall latency improved in the lansoprazole-treated group, while locomotor activity gradually worsened in the DMSO-treated group. At 4 months, locomotion activity was higher in the lansoprazole-treated group than in the DMSO-treated group. **(C)** Western blotting of the ipsilateral and contralateral injected sides of mouse cerebrum homogenates. Insoluble tau bands were higher on the ipsilateral side than on the contralateral side of the brain. **(D)** Quantification bar graph of T46 antibody-positive band intensity. Tau band intensity on the contralateral side was significantly higher in the DMSO-treated group than in the lansoprazole-treated group (*p* = 0.0089). Tau band intensity on the ipsilateral side was also significantly higher in the DMSO-treated group than in the lansoprazole-treated (*p* value 0.0368). IHC, immunohistochemistry; WB, western blotting; Ipsi, ipsilateral; Contra, contralateral; Sar-insol, sarkosyl-insoluble; A68 sol, A68 buffer-soluble; Lan, lansoprazole.

Accelerating rotarod treadmill tests were conducted 0, 1, 2, 3, and 4 months after the stereotactic injection (Figure 1A). Mice were placed on the rotarod treadmill (name) and allowed to acclimate for 2 min. Fall latency was recorded at 40 rpm with an accelerating time of 300 s. The test was repeated five times for each mouse, with a 2-min rest allowed between runs, and their orientation was matched to face forward before each run.

### 2.10 Immunohistochemistry

Four months post-injection, mice were sacrificed and divided into two groups: one for an immunohistochemical analysis and the other for a biochemical analysis. Mice were transcardially perfused with 20 mL cold PBS, followed by 10 mL of 4% paraformaldehyde in PBS. Removed brains were fixed in the same fixative for 24 h. Six-millimeter-thick paraffin-embedded sections were prepared (Hayashi et al., 2021). In tau immunolabeling, sections were permeabilized and endogenous peroxidase was quenched by methanol containing 0.3% H_2_O_2_. To expose antigens, sections were autoclaved in citrate buffer at 120°C for 10 min. The primary antibody, AT8 (specific for tau phosphorylated at Ser202/Thr205, 1:200; Invitrogen), anti-mouse GFAP antibody (1:400, Neomarkers) or anti-Iba1 (specific for microglia, 1:500; Fujifilm Wako Chemicals Japan) were applied at 4°C overnight. With regard to AT8 antibody immunostaining, sections were immersed in simple stain MAX PO (Nichirei Biosciences Inc.) at room temperature for 1 h. With regard to GFAP and Iba1 staining, biotin-conjugated goat anti-rabbit IgG (Vector laboratories) was used as secondary antibody. Sections were stained with the Labeled Streptavidin-Biotin staining methods (VECSTASTAIN ABC Kit, Peroxidase [Standard]). All sections were visualized using the DAB reaction. AT8, GFAP or Iba1-positive areas were quantified using ImageJ software.

### 2.11 Alanine replacement of tau-CTF24

Lys317, Gln351, His362, Lys369, and Lys375 of full-length tau (4R2N) in Paired helical filament (PHF) -tau were predicted to interact with lansoprazole (SwissDock) (Grosdidier et al., 2011). To construct the alanine replacement of Tau-CTF24 at these amino acids, the Toyobo KOD-Plus-Mutagenesis Kit (SMK-101) was used for site-directed mutagenesis with a set of primers (Supplementary Table S3). Alanine-replaced CTF24 was used in place of tau-CTF24 and incubated with lansoprazole. Cells were harvested after a 48-h incubation and soluble and sarkosyl-insoluble tau were subjected to Western blotting.

### 2.12 Statistical analysis

The *t*-test was performed using Microsoft Office Excel 2019 software.

## 3 Results

### 3.1 AD seed-induced insoluble tau in SH-SY5Y cells was reduced by 180 drugs

**Figure 2:**
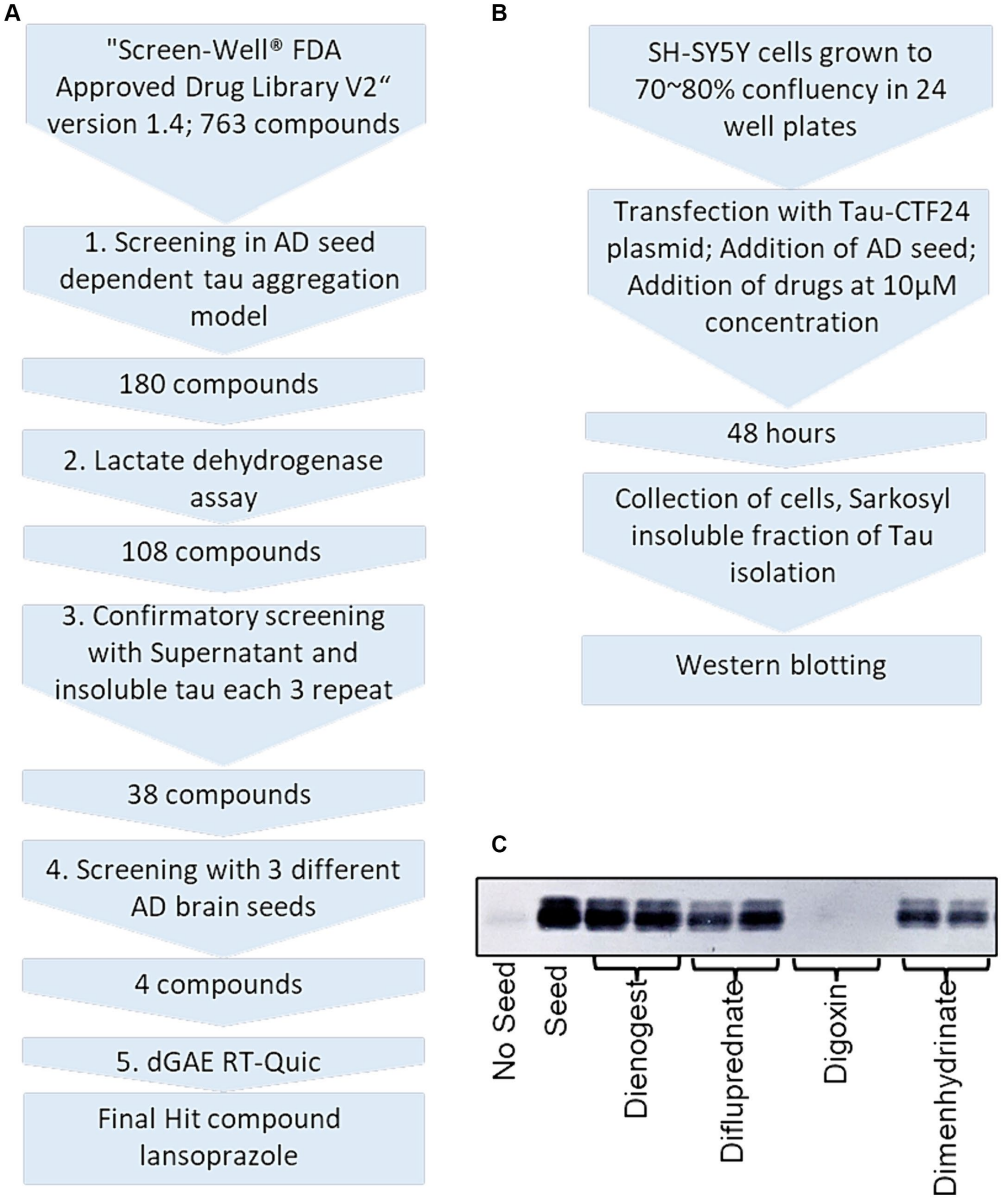
Overview and experimental protocol of FDA-approved drug library screening. **(A)** Five-step procedure of the drug library screening process. **(B)** Alzheimer’s disease seed-dependent cellular model workflow. **(C)** An example of Western blotting of sarkosyl-insoluble tau after drug treatment. Dienogest (synthetic progesterone), difluprednate (corticosteroid), digoxin (Na+/K+ ATPase pump antagonist), and dimenhydrinate (H1-antagonist) were added to SH-SY5Y cells transfected with CTF-24 and the addition of AD seeds. Sarkosyl-insoluble fractions were visualized with the T46 antibody. In this example, only digoxin was a hit compound.

We isolated sarkosyl-insoluble tau seeds from 3 AD patients. In the first screening, SH-SY5Y cells expressing Tau-CTF24 (243–441) induced by AD 1 were treated with 763 compounds (Figures 2A,B). Sarkosyl-insoluble fractions were extracted from cell lysates using A68 buffer. Their amounts were evaluated by Western blotting using the T46 antibody (Figure 2C). Each compound was tested twice and added to one lane each of SDS-PAGE. In this screening, a decrease in at least one lane was considered to indicate a hit compound.

In the first screening, AD seed-induced aggregation was inhibited by 180 drugs (Supplementary Table S4; Supplementary Figure S1). In terms of neurotransmitter-modulating drugs, 19 monoaminergic drugs, such as 6 dopaminergic, 6 serotonergic, 2 adrenergic and 5 histaminergic drugs, were hit compounds. Among the 6 dopaminergic drugs, 4 dopamine receptor-blocking drugs (thioridazine HCl, pimozide, quetiapine fumarate, and aripiprazole) are used as antipsychotics, while 2 dopamine receptor agonists (cabergoline and bromocriptine mesylate) are used to treat Parkinson’s disease. The 6 serotonergic drugs included 4 antidepressants: 3 serotonin reuptake inhibitors (nefazodone-HCl, citalopram HBr, and clozapine) and 1 serotine receptor antagonist (amoxapine). Two serotonin receptor agonists consist of an antimigraine drug (zolmitriptan) and antiemetic drug (granisetron•HCl). Two adrenergic receptor agonists are a bronchodilator [(±)Isoproterenol•HCl] and vasoconstrictor (Epinephrine Bitartrate). Five antihistamine drugs (meclizine dihydrochloride, acrivastine, clemastine fumarate, loratadine, and diphenhydramine•HCl) are allergic drugs, while a first-generation antihistamine drug (hydroxyzine dihydrochloride) is used as an antiemetic drug. Three cholinesterase inhibitors (donepezil HCl, galantamine hydrobromide, and tacrine HCl), which have been prescribed to treat AD, inhibited tau aggregation in SH-SY5Y cells. Two NMDA glutamate receptor antagonists (memantine hydrocholoride and pentamidine isethionate) were identified as hit compounds. Memantine hydrocholoride is used internationally to treat patients with moderate and severe AD.

Eleven immunosuppressive drugs include 6 immunosuppressants containing 2 calcineurin inhibitors (pimecrolimus and cyclosporine A) and 2 mammalian target of rapamycin (mTOR) inhibitors (rapamycin and everolimus). The 5 other immunosuppressive drugs are corticosteroids (an ointment for dermatitis [clobetasol propionate], an asthma medication [beclomethasone dipropionate], and 3 allergy medications [ciclesonide, flunisolide, and mometasone furoate]). Forty-four antineoplastic drugs inhibited insoluble tau. Regarding anti-infectious agents, 10 antibiotics, 15 antifungal drugs, 3 antiprotozoal drugs, 2 antiparasitic drugs, and 8 antiviral drugs were considered to be hit compounds. Among medications for lifestyle-related diseases, 13 antihypertensive drugs, 6 antilipidemic drugs, and 3 antidiabetic drugs were considered to reduce the amount of insoluble tau. Three vitamin D (VD) derivates, 4 contraceptive drugs, and 3 PPI were hit compounds. The classes of the remaining 26 compounds are described as others, antimalarial drugs, disinfectants, and antiarrhythmic drugs in Supplementary Figure S1 (7th and 8th line).

### 3.2 The LDH assay excluded 72 drugs, with 108 remaining

An LDH assay was performed to exclude cytotoxic drugs (Supplementary Figure S2). Donepezil·HCl, a cholinesterase inhibitor, and pindolol, a hypertensive drug, caused most cells to detach and, thus, it was not possible to perform the LDH assay. We considered LDH activity >20% to be cytotoxic. DMSO added wells were considered the baseline LDH activity. Less than baseline was considered to be toxic, as fewer cells than baseline was present at endpoint. Among dopaminergic and serotonergic drugs, only thioridazine-HCl and amoxapine were cytotoxic. Thioridazine was previously shown to exhibit cytotoxicity in a Ca^2+^-dependent manner in HepG2 human hepatocellular carcinoma cells (Chen et al., 2020). Among histaminergic drugs, the LDH activity of H1 receptor antagonists (meclizine dihydrochloride, acrivastine, and clemastine fumarate) was >20%. Clemastine fumarate was previously reported to affect cellular calcium transients via the purinergic receptor P2RX7 to promote inflammasome signaling (Matty et al., 2019). Three of 11 immunosuppressants and 20 of 44 antineoplastic drugs were considered to be cytotoxic. Among anti-infective drugs, 11 of 16 antifungal drugs, all 3 antiprotozoal drugs, and 4 of 8 antiviral drugs were cytotoxic. Four of 13 antihypertensive drugs and 3 of 6 antilipidemic drugs exhibited strong LDH activity. Although statins, inhibitors of 3-hydroxy-3-methylglutaryl-CoA reductase, are widely used as cholesterol-lowering drugs, cytotoxicity in motor neurons has been reported (Bai et al., 2021). Despite their strong LDH activity, 7 drugs (progesterone, rapamycin, everolimus deferasirox auranofin rimantadine, and amiodarone hydrochloride), which were reported to have neuroprotective effect against AD, were not excluded. Progesterone has been shown to exert neuroprotective effects against the pathogenesis of AD (Bassani et al., 2023). Rapamycin and everolimus are mTOR inhibitors and may be involved in autophagy (Oddo, 2012). Since brain iron levels have been associated with AD cognition (Ayton et al., 2020), deferasirox, an iron chelator, may effectively inhibit tau aggregation. In the first screening, auranofin showed a unique ladder band pattern. Rimantadine is an NMDA receptor antagonist that is similar to the anti-AD drug, memantine. Amiodarone hydrochloride has been shown to modulate β-and γ-secretase activities (Mitterreiter et al., 2010).

### 3.3 Soluble tau and insoluble tau analyzes excluded 70 drugs

To identify drugs that effectively inhibit the generation of AD tau filaments, but not tau expression, soluble tau and sarkosyl-insoluble tau were analyzed after the treatment of cells with 108 drugs (Figure 2A; Supplementary Figure S3; Supplementary Table S4). A compound that did not lower the amount of soluble tau, but reduced that of insoluble tau was selected as a hit compound in this step. AD 1 was used as the seed in this step. Each compound was tested 3 times. Among dopaminergic drugs, 2 dopamine receptor blockers (antipsychotics), pimozide and aripiprazole, and the D2 receptor agonist (antiparkinsonian drug), bromocriptine markedly reduced soluble tau. Pimozide was previously shown to reduce toxic forms of tau via 5′ adenosine monophosphate-activated protein kinase-mediated autophagy (Kim et al., 2017). All 5 serotonergic drugs were excluded in this step. The 5-HT2 serotonin antagonist, nefazodone·HCl reduced soluble tau; however, the other 3 serotonin antagonists, citalopram·HBr, clozapine, and granisetron·HCl, did not efficiently reduce insoluble tau. Although zolmitriptan reduced insoluble tau, it is only used for the acute treatment of migraines and is not considered suitable for chronic disease, such as AD; therefore, it was excluded in this step. Although 2 choline esterase inhibitors (drugs for AD) did not reduce soluble tau, the amount of aggregates did not change (Supplementary Figure S3 2nd line). Nine of 25 antineoplastic drugs, 3 of 6 antibiotics, 5 of 6 antifungal drugs, 1 of 2 antiparasitic drugs, and 4 of 5 antiviral drugs reduced soluble tau, possibly due to their cytotoxic effects (Supplementary Figure S3 2nd, 3rd, 4th, and 5th lines). Among antilipidemic drugs, a peroxisome proliferator-activated receptor type alpha (PPARα) activator (fenofibrate) and 2 statins (atorvastatin hemicalcium salt and rosuvastatin calcium) reduced soluble tau. At the end of the third screening, 38 drugs were selected.

### 3.4 Thirty-eight drugs were replicated in 3 different AD seeds and 4 drugs were selected

**Figure 3:**
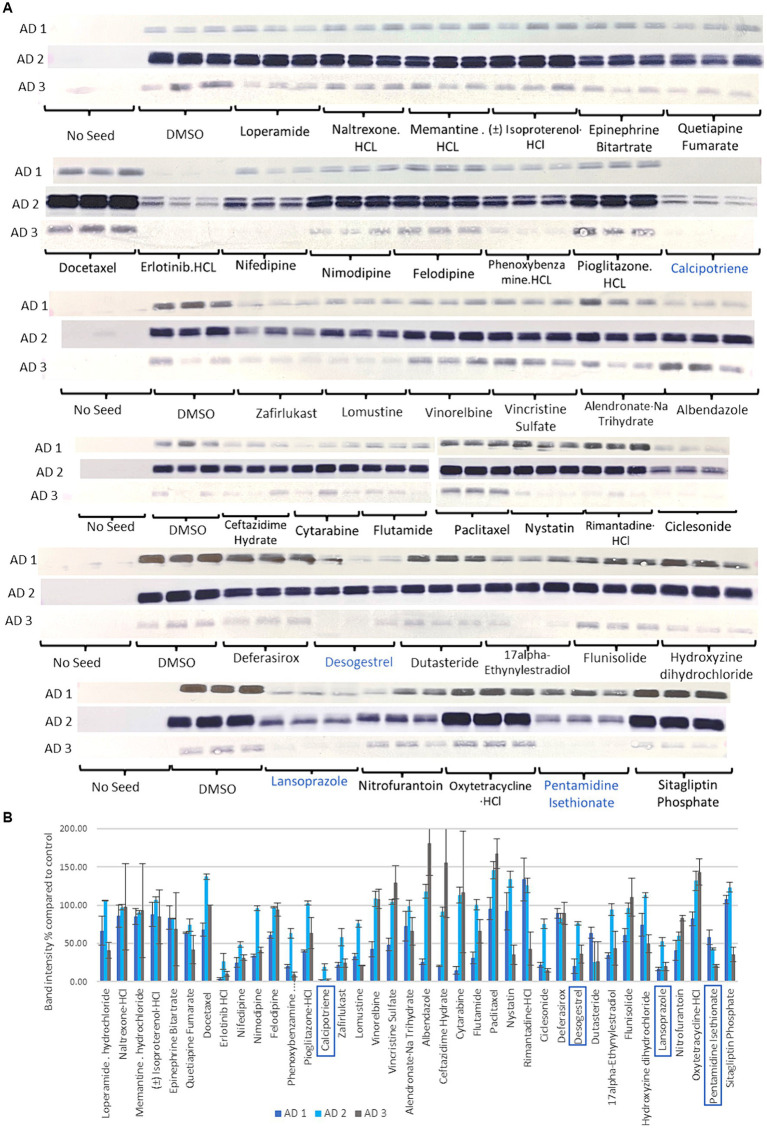
Sarkosyl-insoluble tau analysis of 38 selected compounds in the third step using multiple AD seeds. **(A)** Western blot results after compounds treatment. Control blotting localized on the left of the 1st, 3rd, 4th, 5th, and 6th lines is shown. No seed showed any band, while the DMSO-added control showed positive bands. Seeding activity was higher in AD 2 than in AD 1 and AD 3. Calcipotriene, desogestrel, lansoprazole, and pentamidine isethionate in the green line were selected. **(B)** Quantification of T46 band intensity in Western blotting with 3 different AD seeds. Four compounds with the lowest band intensities, calcipotriene, desogestrel, lansoprazole, and pentamidine isethionate, were selected in this step. Although erlotinib exhibited the lowest activity, the antineoplastic drug was excluded.

Tau seeding activity varies across patients with AD (Dujardin et al., 2020). Therefore, the effectiveness of the inhibition of tau aggregation was tested with 3 different AD seeds (AD 1, AD 2, and AD 3). Thirty-eight compounds were tested 3 times for the 3 AD seeds (Figure 3). While the seeding capacities of AD 1 and AD 3 were similar, that of AD 2 was higher (Figure 3). Treatments with erlotinib HCl, calcipotriene, lomustine, desogestrel, dutasteride, lansoprazole, and pentamidine isethionate reduced insoluble tau to a similar extent in all 3 AD seeds. In consideration of drug repositioning for AD, the antineoplastic drugs, erlotinib HCl and lomustine were excluded. The Vitamin D derivate, calcipotriene, the PPI, lansoprazole, the contraceptive drug, desogestrel, and the NMDA receptor antagonist, pentamidine isethionate were selected for subsequent experiments.

### 3.5 Lansoprazole and calcipotriene inhibited the AD seed-induced RT-QuIC reaction

**Figure 4:**
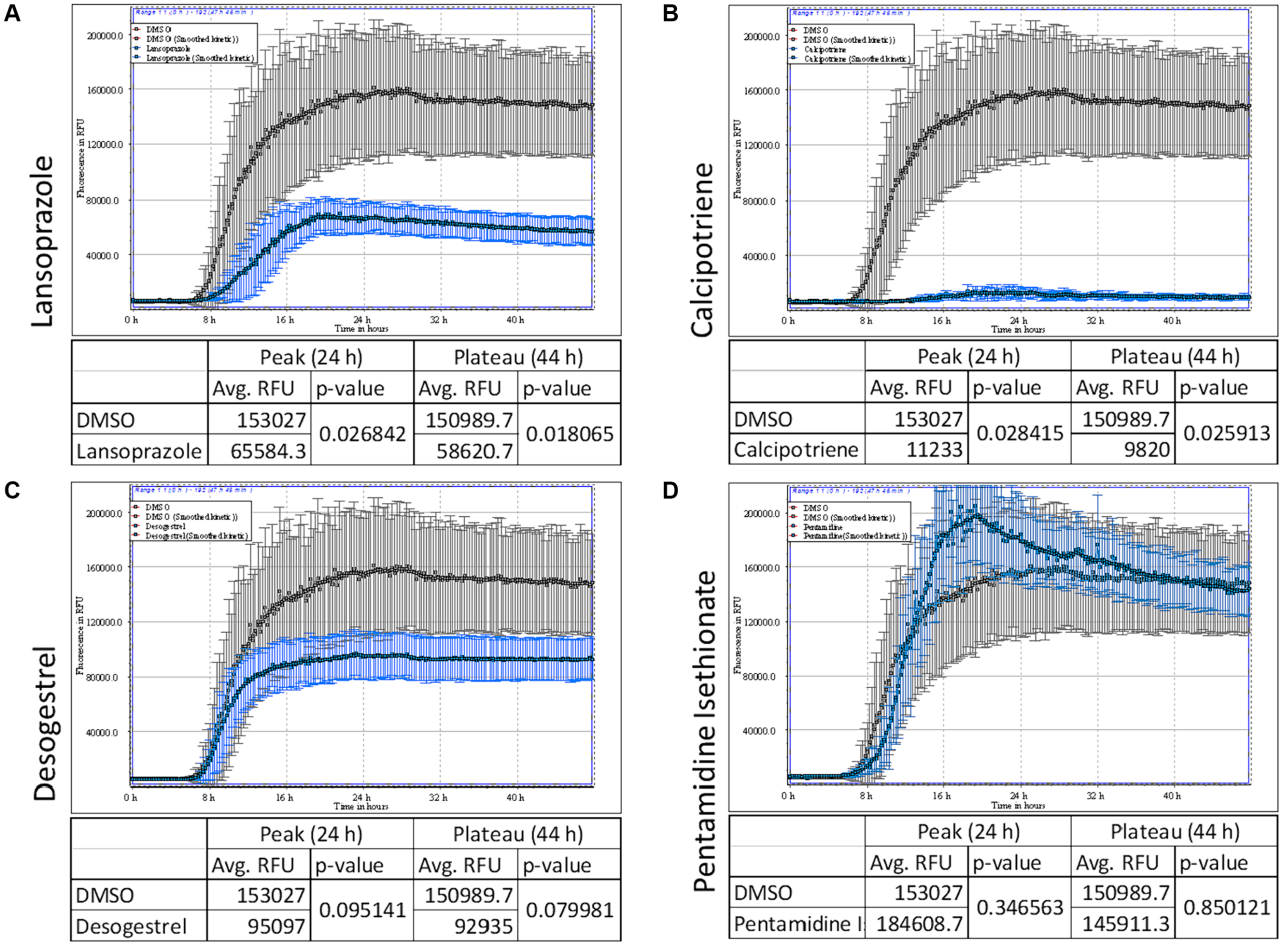
AD seed-induced real-time quaking-induced conversion (RT-QuIC). ThT fluorescence curves of 7.5 μM tau dGAE contained with 50 μM lansoprazole **(A)**, calcipotriene **(B)**, desogestrel **(C)** and pentamidine isethionate **(D)**. Vertical lines indicate ThT fluorescence intensity, and the horizontal line denotes time. Compounds were added at a concentration of 50 μM. Lansoprazole and calcipotriene reduced ThT fluorescence, whereas pentamidine and desogestrel did not. In each panel, DMSO is denoted in gray and test compounds are denoted in blue.

To identify drugs that effectively prevent tau aggregation, calcipotriene, lansoprazole, desogestrel, and pentamidine isethionate were co-incubated with dGAE tau (Figure 4). Drugs were added at a concentration of 50 μM. The ThT fluorescence curve showed that lansoprazole and calcipotriene significantly inhibited AD seed-induced dGAE aggregation after 24 and 48 h, whereas pentamidine isethionate did not (Figures 4A–D). Calcipotriene was the most effective inhibitor of ThT fluorescence, followed by lansoprazole. These results indicate that lansoprazole and calcipotriene directly interacted with tau dGAE and suggest the potential of their application to AD propagation mouse models.

### 3.6 Lansoprazole improved locomotion in AD seed-injected mice

AD2 seeding activity was the highest between 3 AD patients in the third screening (Figure 3). AD2 seed-derived sarkosyl-insoluble tau was injected into the right caudate putamen of 25 C57BL6/J wild-type female mice. After a 1-week healing period, the rotarod test was performed and the intranasal administration of drugs (100 ng per day) or 10 μL DMSO was initiated (Figure 1A). Calcipotriene-injected mice showed significant weight loss after 7 days and treatment was discontinued (data not shown). After 3 months, locomotor activity in the rotarod treadmill test was significantly higher in lansoprazole-treated mice than in DMSO-treated mice (Figure 1B). This effect continued at 4 months.

### 3.7 Lansoprazole decreased tau pathology in AD seed-injected mice

Four months post-injection, mice were sacrificed by cervical dislocation. The cerebrums of 3 DMSO-treated mice and 3 lansoprazole-treated mice were dissected into 2 hemispheres and rapidly frozen in liquid nitrogen. A biochemical analysis demonstrated that the amounts of sarkosyl-insoluble tau in all 6 mice were higher on the ipsilateral side than on the contralateral side of the brain, while soluble tau bands did not significantly differ (Figure 1C). In comparisons of sarkosyl-insoluble band intensities between DMSO (*n* = 3)- and lansoprazole (*n* = 3)-treated mice, the lansoprazole group showed significantly lower sarkosyl-insoluble tau levels on both the ipsilateral and contralateral sides of the brain (Figure 1D).

**Figure 5:**
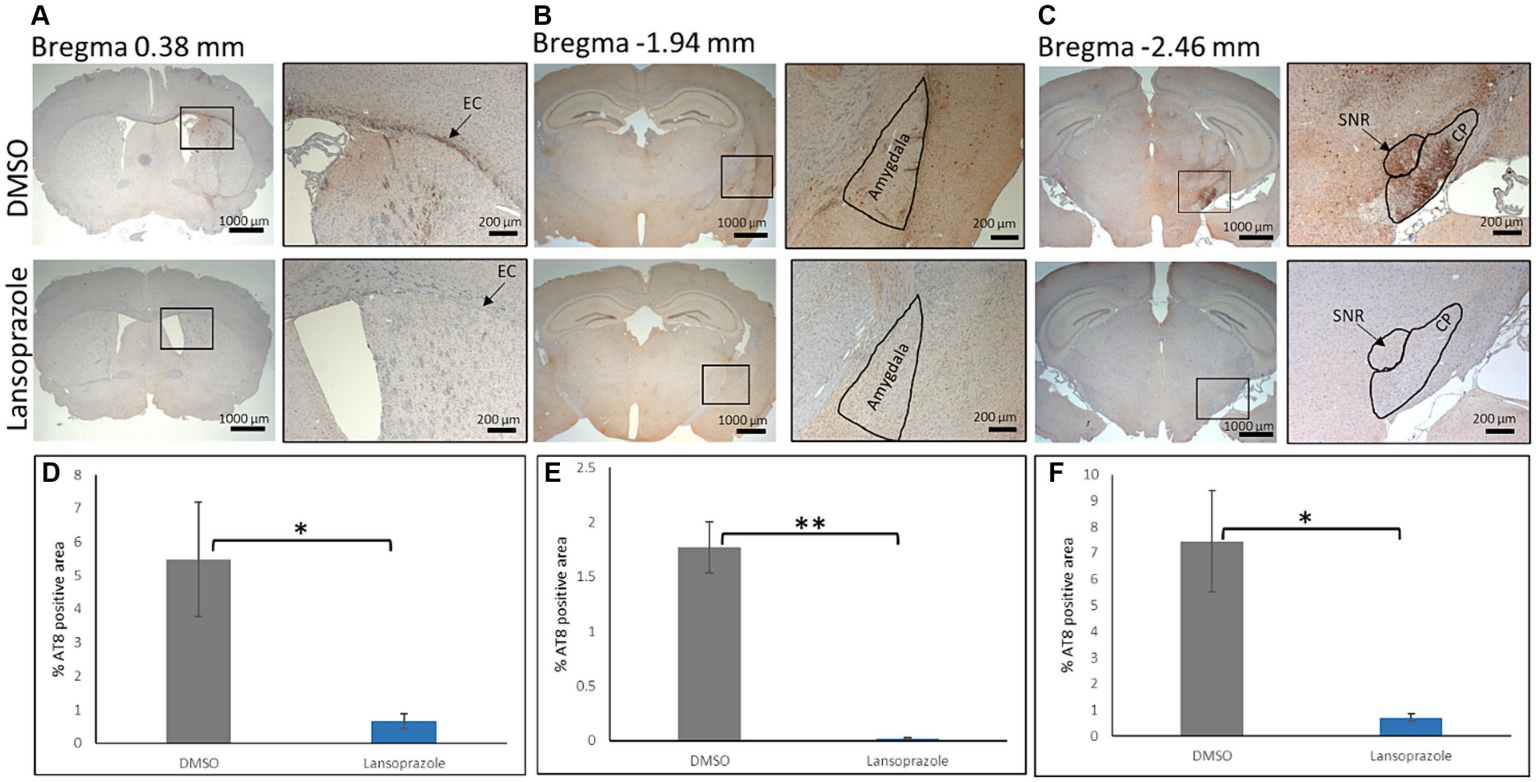
Lansoprazole treatment attenuated the phosphorylated tau pathology. At the bregma 0.38 mm **(A)**, the external capsule (EC) showed the highest AT8 immunoreactivity on the ipsilateral side. At the bregma-1.94 mm **(B)**, the amygdala showed strong AT8 immunoreactivity in the DMSO-treated group. At the bregma-2.46 mm **(C)**, the substantia nigra, reticular region (SNR), and cerebral peduncle (CP) showed strong AT8 immunoreactivity. **(D–F)** show quantification bar graphs of the percentage of AT8-positive areas in the EC and caudate putamen **(A)**, amygdala **(B)**, and **(C)** (CP and SNR) respectively. AT-positive areas in the EC, SNR and CP were smaller in the lansoprazole-treated group. Insets in the left panels represent the right panels in **(A–C)** * indicates *p* < 0.05, ** indicates *p* < 0.01 Bar = 200 mm.

To perform an immunohistochemical analysis, mice were sacrificed by perfusion with ice-cold PBS and fixed with 4% PFA in PBS. Paraffin-embedded brain sections were prepared at the +0.38 mm, −1.94 mm, and-2.46 mm positions (Figure 5). In all mice on the ipsilateral side, AT8-positive lesions showed higher intensity and greater areas when compared to the contralateral side. At the bregma 0.38 level, the external capsule (EC) had the highest AT8 immunoreactivity. In comparisons with AT8 immunoreactivity in DMSO-treated mice (*n* = 3), lansoprazole-treated mice (*n* = 3) showed a significant reduction in AT8-positive lesions in the EC and caudate putamen (Figures 5A,D). At the bregma-1.94 level, the ipsilateral amygdala showed strong immunoreactivity for AT8. The AT8-positive area was larger in the DMSO-treated group than in the lansoprazole-treated group (Figures 5B,E). At the bregma-2.46 level in DMSO-treated mice, very strong AT8-positive lesions were observed at the substantia nigra, reticular part (SNR), and cerebral peduncle (CP). In these regions, a significant reduction in tau lesions was also noted in lansoprazole-treated mice (Figures 5C,F).

To investigate the involvement of brain inflammatory cells, GFAP-positive astrocytes and Iba1-positive microglia were counted in the EC and caudate putamen. No significant difference was noted between DMSO-treated and lansoprazole-treated mice (Supplementary Figure S4).

### 3.8 Gln351, His362, and Lys369 interact with lansoprazole

**Figure 6:**
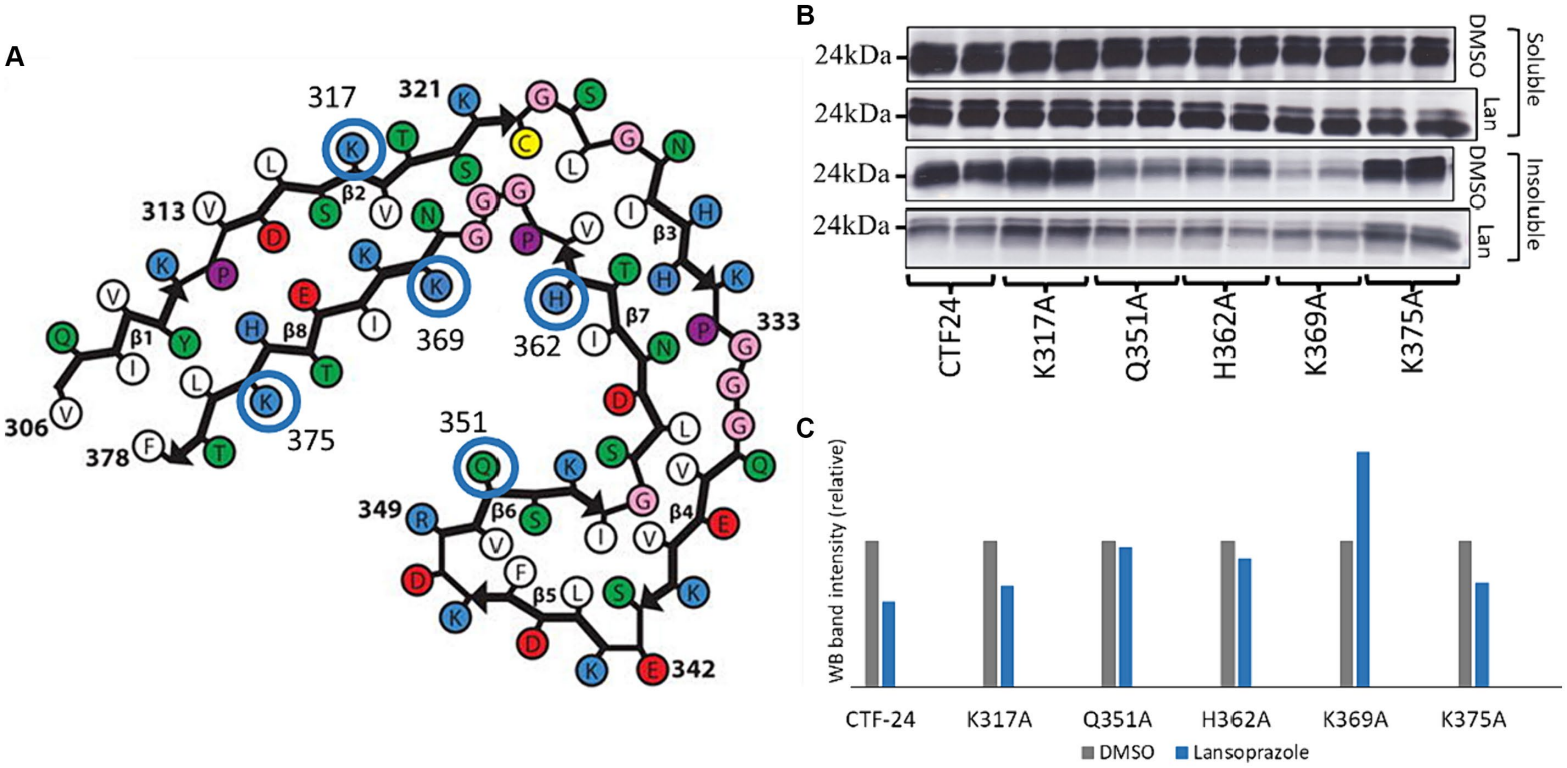
Lansoprazole effects on tau aggregation of alanine replaced mutants at 5 predicted binding sites of predicted binding sites (K317, Q351, H362, K369, and K375). **(A)** Predicted binding sites of lansoprazole and tau paired helical filaments of AD brains (Fitzpatrick et al., 2017) in SwissDock. **(B)** Western blotting of alanine-replaced Tau-CTF24 with the T46 antibody. **(C)** Relative band intensity of insoluble tau extracted from lansoprazole-treated cells compared to DMSO. Lansoprazole effects on tau aggregation with K317A and K375A Tau-CTF24 were clearly observed, while Q351A, H362A, and K369A replacements resulted in similar lansoprazole effects on tau aggregation **(B,C)**. This indicates that these amino acids are essential for binding with lansoprazole; Lan, lansoprazole.

To elucidate how lansoprazole interacts with AD tau, we used the SwissDock online docking web server (Grosdidier et al., 2011). The website predicted that lansoprazole potentially interacts with the Lys317, Gln351, His362, Lys369, and Lys375 residues of the AD tau common protofilament core (6HRE; PHF from sporadic AD [PROTEIN DATA BANK: PDB]) (Fitzpatrick et al., 2017) (Figure 6A; Supplementary Figure S5). Mutated plasmids with alanine replaced at these sites were used instead of Tau-CTF24 to identify the amino acids that lansoprazole interacts with. The absence of a reduction in aggregation with mutated Tau-CTF-24 indicated that the original amino acid was essential for lansoprazole binding. After a 48-h incubation, lansoprazole reduced the insoluble band of Tau-CTF24 with no mutation. The K317A and K375A mutations did not impair the effects of lansoprazole. Although Q351A-, H362A-, and K369A-mutated Tau-CTF-24 were not recruited to the same extent as control Tau-CTF24, the treatment with lansoprazole did not reduce insoluble tau bands (Figures 6B,C). These results indicate that lansoprazole bound to C-shaped AD tau filaments at Q351A, H362A, and K369A on the inner side of the cavity.

## 4 Discussion

In the present study on drug repositioning for AD, we searched 763 drugs in the Screen-Well^®^ FDA-Approved Drug Library V2 using an AD seed-dependent cell model. In the first screening, 180 drugs were considered to reduce insoluble tau induced by 1 AD seed in SH-SY5Y cells. In the second screening, cytotoxic drugs were excluded using the LDH assay and 108 drugs were selected. In the third screening, drugs that decreased soluble tau, but not insoluble tau were excluded and 38 drugs were selected. In the fourth screening, multiple AD seeds were used and the analysis identified 4 drugs: lansoprazole, desogestrel, calcipotriene, and pentamidine isethionate. In the *in vitro* cell-free assay, RT-QuIC showed that calcipotriene and lansoprazole were more effective than pentamidine isethionate and desogestrel. An intranasal treatment with lansoprazole effectively improved locomotor activity, attenuated the phosphorylated tau-positive pathology, and reduced insoluble tau in the *in vivo* AD seed-injected mouse model. These results indicate that the widely-used PPI, lansoprazole, effectively inhibited AD seed-dependent tau aggregation in cells, *in vitro*, and *in vivo*.

The inhibition of tau aggregation by lansoprazole may be related to its direct effects on tau assembly formation rather than the mechanism of action of a PPI. Four PPI were included in the Screen-Well® FDA-Approved Drug Library V2. They inhibit the H+, K + ATPase of gastric cells and suppress gastric acid secretion. In the present study, omeprazole and pantoprazole did not affect tau aggregation (data not shown), while rabeprazole markedly reduced soluble tau in the third screening (Supplementary Figure S3). In the *in vitro* cell-free assay, RT-QuIC also demonstrated that lansoprazole markedly reduced tau aggregation. These results suggest that lansoprazole directly bound to tau and suppressed tau filament formation. Previous studies also indicated that lansoprazole bound to heparin-induced tau filaments and PHFs isolated from AD patients and, thus, it is regarded as a good candidate for a tau PET tracer (Rojo et al., 2010). The novel tau imaging reagent, [18F]N-methyl lansoprazole showed good brain uptake in healthy controls; however, its retention in brains with mild cognitive impairment/AD and progressive supranuclear palsy was low (Kramer et al., 2020). Predictions of molecular interactions at SwissDock showed that binding sites with the most favorable Gibbs free energy for binding in the AD tau protofilament core (306–378 of the tau protein) (Fitzpatrick et al., 2017) were Lys317, Gln351, His362, Lys369, and K375. Even without lansoprazole treatment, cells expressing Tau-CTF24 with alanine replacement at Q351, H362 or K369 have reduced the amount of insoluble tau (Figure 6B). Histidine (H) and lysine (K) have positively charged side chains and glutamine (Q) is a polar amino acid. Since it is known that the rate of assembly of the tau protein into filaments is greatly enhanced by polyanions (Friedhoff et al., 1998), it is probable that replacement of positively charged amino acids or a polar amino acid with non-charged or non-polar amino acid may result in reduced tau assembly. The analysis of cryo-electron microscopy demonstrated that the core of corticobasal degeneration (CBD) filament adopts four-layered fold which encloses a large nonproteinaceous densities (Arakhamia et al., 2020; Zhang W. et al., 2020). It is speculated that the densities are polyanionic molecules, partly because they are surrounded by the positively charged side chains of K290, K294 and K370. Montgomery et al. recently measured the ability of 37 diverse anionic biomolecules to initiate tau aggregation and they proved that crucial role of polyanion-tau interactions in modulating tau conformational dynamics (Montgomery et al., 2023). In the present study, Q351A, H362A, and K369A mutations reduced overall tau aggregation efficacy by lansoprazole, indicating the inner side of the C-shaped AD protofilament (Figure 6). The cryo-EM structures of AD tau filaments with the PET ligand APN-1607 identified two major sites in the groove between the side chains of Q351 and K353 in the straight filament (SF) and PHFs and a third major site in the C-shaped cavity of SF (Shi et al., 2021). Interestingly, our mutation study also demonstrated that Q351 was a predicted site to interact between lansoprazole and the tau protein. Lansoprazole and APN-1607 have the structural similarities in those two heterocyclic compounds, such as benzimidazole and pyridine in lansoprazole and benzothiazole and pyrimidine rings in APN-1607, are bound by a spacer. It is speculated that APN-1697 forms a hydrogen-bonded ladder along the filament axis with Q351 (Shi et al., 2021). Since lansoprazole has a sulfoxide which has a negative charge or binds with hydrogen bond, it would be bound to Q351 like APN-1697.

Another mechanism of action of lansoprazole is as an agonist of liver X receptors (LXRs), which are members of the nuclear receptor superfamily that are canonically activated by oxidized derivates of cholesterol (Cronican et al., 2010). LXRs increased Abca1 and apoE protein levels in primary astrocytes and an LXR agonist reduced the accumulation of amyloid β *in vitro* and *in vivo* (Cao et al., 2007; Cronican et al., 2010; Bunay et al., 2021). Furthermore, a treatment with lansoprazole attenuated streptozotocin-and high fat diet (HFD)-induced memory deficits in mice accompanied with restoring acetylcholinesterase activity, and reducing inflammation markers, including thiobarbituric acid reactive species and myeloperoxidase, in the brain (Sodhi and Singh, 2013). Since a phenotypic analysis of LXR gene-modified mice revealed various diseases, including diabetes, obesity, inflammatory responses, Parkinson’s disease, and AD, LXR drug discovery has been attempted (Bunay et al., 2021). The administration of the LXR receptor agonist T0901317 to APP/PS1 double transgenic mice improved HFD-induced learning deficits, reduced the expression of caveolin-1, and decreased the production of brain amyloid β (Luo et al., 2022).

In our study, lansoprazole demonstrated a significant reduction in AT8-positive tau immunoreactivity in the brains of mice. AT8 antibody is a well-characterized mouse monoclonal antibody specifically targeting phosphorylation sites at Serine 202 and Threonine 205 (using the numbering of longest human brain tau isoform) (Goedert et al., 1995). Previous research has highlighted the involvement of mitogen-activated protein kinases (MAPKs), specifically p38 MAPK, extracellular signal-regulated kinase 2 (ERK2) and cyclin-dependent kinase 5 (cdk5) in the phosphorylation of tau at these sites (Reynolds et al., 2000; Kimura et al., 2018). Cao et al. (2013) demonstrated that the inhibition of p38 MAPK activity with SB203580 abolished tau hyperphosphorylation in neurons. Additionally, miR-483-5p-mediated repression of ERK1/2 has been associated with reduced phosphorylation of tau at S202 (Nagaraj et al., 2021). Previous investigations by Koshio et al. suggested that lansoprazole may suppress the signal transduction pathways mediated by ERK1/2 and p38 MAPK (Koshio et al., 2010). Furthermore, lansoprazole has been implicated in modulating intracellular signaling cascades beyond MAPK pathways. In renal cells, lansoprazole has been shown to inhibit mTOR/S6K and ERK signaling proteins (Nantavishit et al., 2018). Collectively, these findings suggest that lansoprazole may exert its effect on reducing AT8-positive tau through the inhibition of p38 MAPK and ERK1/2 signaling pathways.

PPI are the first-line treatment for gastroesophageal reflux disease and peptic ulcers (Namazi and Jowkar, 2008). With the increasing use of PPI, safety issues have been raised. Epidemiological research on the relationship between the use of PPI and the risk of dementia has produced conflicting findings. A meta-analysis of six studies from 2014 to 2018 revealed a significant increase in the risk of dementia (Zhang Y. et al., 2020). A recent community-based cohort from 1987 to 2017 also showed that PPI users were at a higher risk of developing dementia (Northuis et al., 2023). In contrast, a double-blind cohort trial of 17,598 participants assigned to groups given pantoprazole or placebo reported no significant difference in the risk of developing dementia (Moayyedi et al., 2019). These discrepancies may be partly attributed to large-scale epidemiological studies generally including participants taking PPI. PPI are divided into first-generation PPI (omeprazole, lansoprazole, and pantoprazole) and second-generation PPI (rabeprazole, ilaprazole, and astemizole). In the present study, only lansoprazole decreased tau aggregation, while omeprazole, pantoprazole, and rabeprazole did not affect tau aggregation.

Calcipotriene effectively reduced AD seed-induced tau aggregation in SY-SY5Y cells (Figure 4) and RT-QuIC (Figure 1). Calcipotriene, a synthetic VD analog, is used as a dermatological ointment against psoriasis and it induces the differentiation of keratinocytes and suppresses their proliferation, thereby reversing the abnormal keratinocyte changes associated with psoriasis. VD is a neuroprotective steroid that regulates multiple pathways that are important for mature brain function. VD induces its genomic effects through its nuclear receptor, the VD receptor (VDR). VD improved learning abilities in an AD rat model by regulating the VDR/ERK1/2 signaling pathway (Bao et al., 2020). VD also decreased total tau and phosphorylated tau levels in an okadaic acid-treated AD mouse model and in SH-SY5Y cells with rescued methylated PP2A by increasing the expression of leucine carboxyl methyltransferase 1 and 5,10-methylenetetrahydrofolate reductase (Pan et al., 2021). However, the intranasal administration of 10 μL calcipotriene to mice at 20 mg/mL for 7 days reduced body weight to 15–16 g from 24 to 25 g, becoming less active, losing fur, and generally appearing sick.

All 38 compounds identified in the third screening have the potential to be AD drug candidates (Figures 3A,B). The present study focused on the four most effective compounds due to scope constraints. Regarding the iron chelator, deferasirox (Figure 3 7th line), previous studies demonstrated that iron overload directly correlated with cognitive decline in AD, and ferroptosis, a type or programmed cell death process characterized by iron overload and triggered by lipid peroxidase, has been shown to be involved in the generation of Ab plaques and tau accumulation in AD (Wang et al., 2022). Deferasirox administered orally thrice weekly slightly decreased phosphorylated tau without improving memory in Tg2576 mice overexpressing a mutant human APP protein and JNPL3 mice overexpressing a mutant human tau protein (Kwan et al., 2022). Another iron chelator, deferiprone decreased brain iron levels and sarkosyl-insoluble tau and this was accompanied by improvements in anxiety-like behavior in a tauopathy model [rTg(tauP301L)4,510] (Rao et al., 2020). In terms of two adrenergic drugs, (±) isoproterenol·HCl and epinephrine bitartrate (Figure 3A 1st line), isoproterenol inhibited the formation of toxic tau granular oligomers (Ikenuma et al., 2019) and epinephrine reduced tau aggregation with the disruption of tau R3-R4 protofibrils (Zou et al., 2022). Memantine HCl (Figure 3A 1st line), an anti-dementia drug, and quetiapine fumarate (Figure 3A 1^st^ line), an antipsychotic, are widely used in the treatment of patients with AD. Both drugs are capable of inhibiting AD seed-dependent tau aggregation, which encourages their prescription by physicians. Three microtubule-targeting agents (docetaxel [Figure 3A 2nd line], vincristine sulfate [Figure 3A 3rd line], and paclitaxel [Figure 3A 4th line]) promotes assembly of microtubules from tubulin and hyperstabilizes microtubules. A microtubule bundling experiment showed that microtubule doublets and triplets with increasing tau were present at a low paclitaxel concentration, and this condition may decrease intracellular free tau and tau aggregation (Choi et al., 2017). Among diabetic drugs, the PPARα agonist, pioglitazone·HCl (Figure 3A 6th line) prevented tau oligomerization in wild-type tau-overexpressing M1C cells (Hamano et al., 2016), while the dipeptidyl peptidase-4 (DPP-4) inhibitor, sitagliptin phosphate (Figure 3A 6th line), increased tau phosphorylation in rats and primary neurons (Kim et al., 2012). Furthermore, the novel DPP-4 inhibitor, Gramcyclin A, inhibited the phosphorylation of insulin receptor-1 and tau phosphorylation in APP/PS1/tau triple transgenic mice (Li et al., 2022). Among antihypertensive drugs, three Ca channel blockers, nifedipine, nimodipine, and felodipine, decreased insoluble tau (Figure 3A 2nd line). Mitogen-activated protein kinase phosphorylated tau and activated the L-voltage-sensitive calcium channels (Ekinci et al., 2003). In this respect, Ca channel blockers inhibit tau phosphorylation and aggregation. Regarding the two estrogen stimulants, desogestrel and 17α-ethinylestradiol (Figure 3A 5th line), estrogen prevented caspase-3-mediated tau cleavage and decreased tau aggregation in optic nerve head astrocytes (Means et al., 2021).

Previous studies reported small-molecule tau aggregation inhibitors among FDA-approved drugs. The results are not consistent with the present study. Rifampicin, an antituberculosis agent, effectively reduced neurotoxic oligomers under cell-free conditions, reduced tau hyperphosphorylation and improved cognition in APPosk mice (Aβ oligomer model), Tg2576 mice, and tau609 mice (Umeda et al., 2016). However, in the present study, it did not reduce the amount of insoluble tau in the first screening (data not shown). R-(−)-apomorphine, a non-selective dopamine agonist, and raloxifene, an antihypocalcemic bone density conservation agent, were found to be phosphorylated tau aggregation inhibitors, but did not exert any effect in the first screening (data not shown) (Liu et al., 2020). HCS using N2a cells expressing the pro-aggregant repeat domain of tau selected 18 inhibitors from a chemogenomic library of 1,649 compounds. Two inhibitors, ivermectin, a chloride channel (GluCl) activator, and spironolactone, an androgen receptor, were included in the FDA-drug library used in the present study (Pickhardt et al., 2019). Ivermectin, an antiprotozoal drug, decreased the amount of insoluble tau in the first screening (Supplementary Figure S1, 5th line), but exhibited high LDH toxicity and, thus, was excluded in the second screening (Supplementary Figure S1, 3rd line), while spironolactone did not affect tau aggregation in the first screening (data not shown). In a rat cortical neuron assay induced by AD brain seeds, 13 compounds from a library of mostly approved drugs inhibited tau aggregation (Crowe et al., 2020). Four of the 13 compounds, atracurium and cisatracurium, acetylcholine receptor antagonists, metoclopramide, a D2 receptor antagonist, and tacrine, an acetylcholine esterase inhibitor, were included in our library. Atracurium, cisatracurium, and metoclopramide did not affect tau aggregation in the first screening (data not shown), while tacrine tau aggregation was not sufficient and, thus, it was excluded in the third screening (Supplementary Figure S3 2nd line). Since the present study used AD brain seed-induced models, the effects of the identified compounds on tau aggregation are likely to be different.

Aside from FDA-approved drugs, basic research confirmed that some small-molecule tau aggregation inhibitors were effective and they reached the stage of clinical trials (Congdon et al., 2023). Curcumin was originally identified as a phenolic compound that inhibited Aβ amyloid aggregation (Dhouafli et al., 2018), and it also reduced tau aggregation and improved cognition in human tau transgenic mice (Ma et al., 2013). A second phase II study on curcumin was conducted and scheduled for completion in 2020; however, its findings have yet to be released (Congdon et al., 2023). Methylene blue/LMTX crosses the blood–brain barrier and was shown to reduce insoluble tau and improve learning and memory in mice expressing full-length pro-aggregant human tau (Hochgrafe et al., 2015). Although several phase III trials on the effects of this drug in patients with AD have been completed, none produced positive findings (Congdon et al., 2023). Another phase III trial on LMTX is still continuing. Tau forms a number of assemblies, including soluble oligomers, insoluble filaments, and liquid droplets (Umeda et al., 2016). Intermediated of epigallocatechin gallate, a well-known tau aggregation inhibitor, bound to tau fibrils was cryogenically trapped and the use of this pharmacophore in the *in silico* screening of a library of drug-like small molecules discovered several candidates (Seidler et al., 2022).

Despite the significant results obtained in the present study, there are a number of limitations that need to be addressed. The uniform concentration (10 μM) used for all compounds in cellular experiments may not account for individual compound variations. Some effective drugs at lower concentration may exhibit cytotoxicity and promote aggregation at 10 uM concentration. Additionally, injecting AD brain-derived tau fibrils into the caudate putamen, instead of the hippocampus, poses a limitation, albeit it was a strategic choice based on the larger phosphorylated tau-positive area observed in our pilot studies. Due to the short duration of treatment in mice, although without adverse effects, the long-term impact of the intranasal administration of lansoprazole remains unknown. Future investigations that address these limitations will provide a more comprehensive understanding of the potential clinical application of these results.

We herein demonstrated that lansoprazole effectively reduced AD seed-dependent tau aggregation in SH-SY5Y cells, *in vitro* tau filament formation, and a mouse model. Lansoprazole has been shown to penetrate the blood–brain barrier in mice (Rojo et al., 2010) and human healthy controls and AD patients (Kramer et al., 2020). Since lansoprazole is generally safe for long-term use and an FDA-approved drug, it is a promising candidate for the treatment of AD.

## Data availability statement

The original contributions presented in the study are included in the article/Supplementary materials, further inquiries can be directed to the corresponding author.

## Ethics statement

The studies involving humans were approved by the Ethics Committee of Juntendo University School of Medicine (approval number: 2012068). The studies were conducted in accordance with the local legislation and institutional requirements. The participants provided their written informed consent to participate in this study. The animal study was approved by the committee for Animal Experiments of Juntendo University (Permit number: 2023157). The study was conducted in accordance with the local legislation and institutional requirements.

## Author contributions

AI: Data curation, Formal analysis, Visualization, Writing – original draft, Writing – review & editing. SS: Investigation, Methodology, Writing – review & editing. MU: Methodology, Supervision, Writing – review & editing. ME: Supervision, Writing – review & editing. KI: Data curation, Supervision, Validation, Writing – review & editing. MH: Methodology, Writing – review & editing. NH: Funding acquisition, Supervision, Writing – review & editing. YM: Conceptualization, Data curation, Funding acquisition, Investigation, Supervision, Validation, Visualization, Writing – original draft, Writing – review & editing.
