# Supplementary material for: Selection of lansoprazole from an FDA-approved drug library to inhibit the Alzheimer’s disease seed-dependent formation of tau aggregates
Source: Front Aging Neurosci. 2024 Mar 26;16:1368291. doi: 10.3389/fnagi.2024.1368291 (PMC11022852; doi:10.3389/fnagi.2024.1368291)
Supplement: Supplementary file 1 [file Data_Sheet_1.docx]

Supplementary Material

# Supplementary Figures


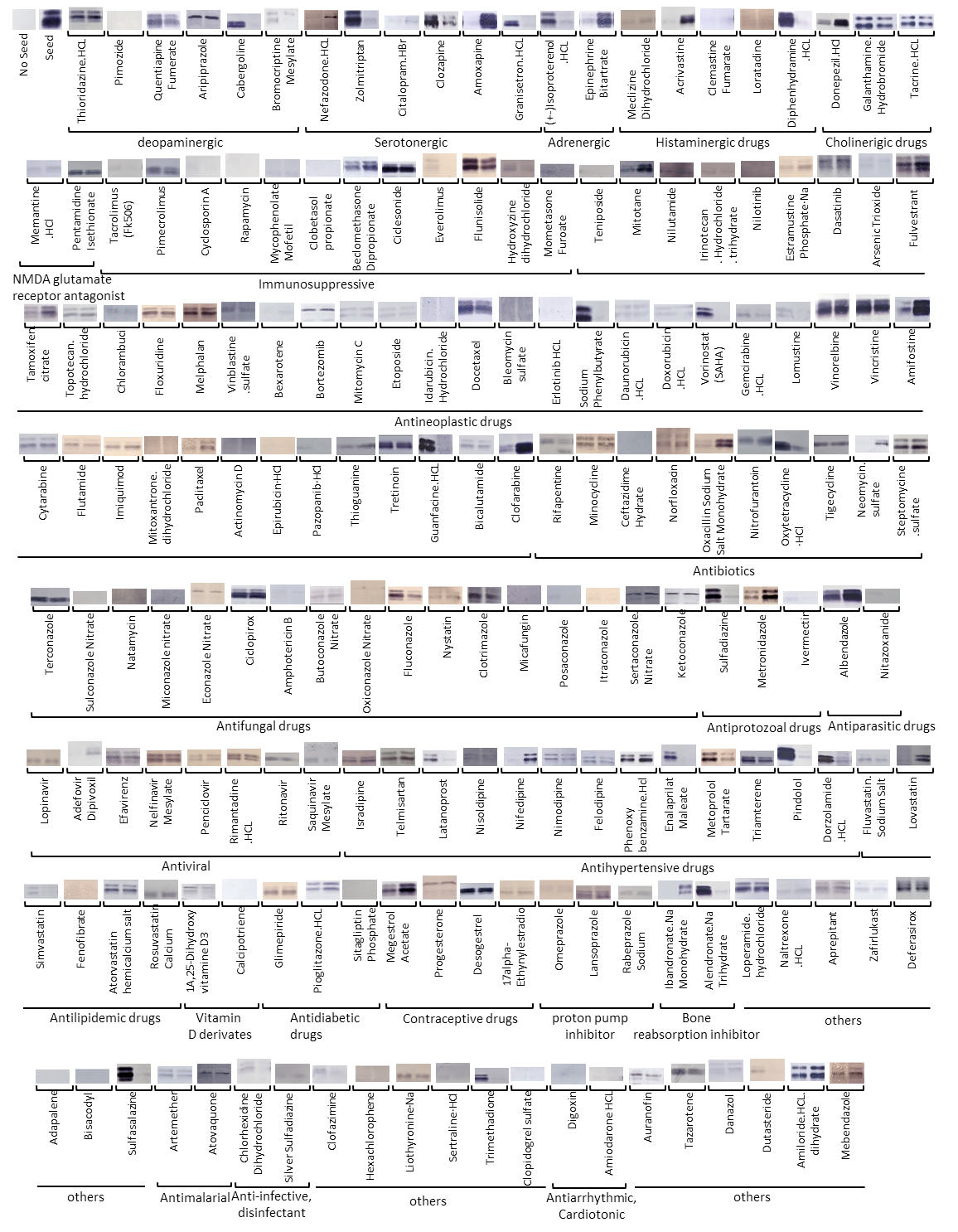


**Supplementary Figure 1.** The western blotting bands of 180 drugs selected at the primary screening. Sarkosyl-insoluble fractions after each drug addition were extracted and visualized with T46 antibody. At this step, the decrease in at least the one lane is considered to be a hit compound.


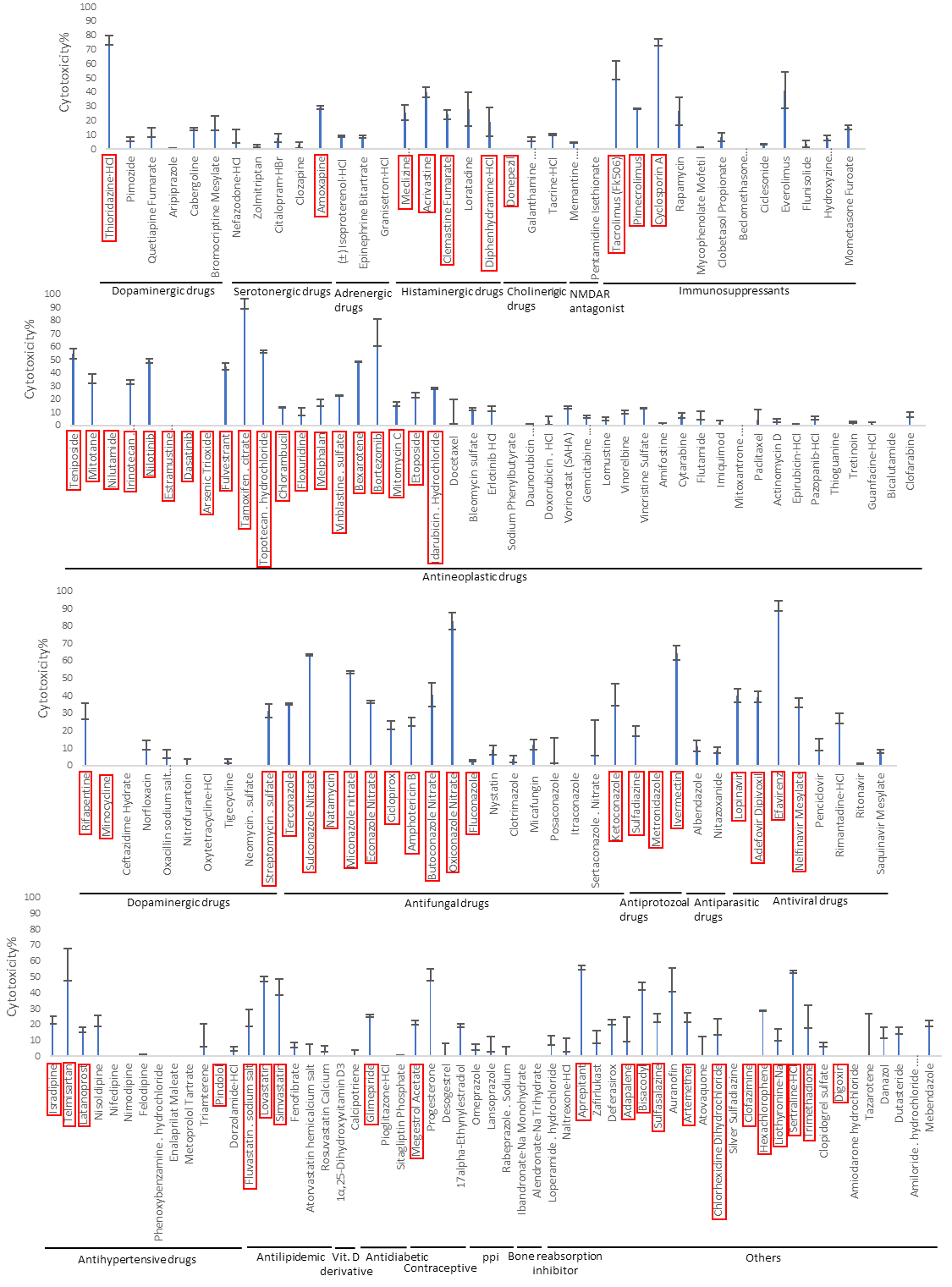


**Supplementary Figure 2.** The results of LDH assay for 180 drugs. LDH assay excluded 72 drugs. We consider that over 20 % LDH activity as cytotoxic. Drugs surrounded in the red line were excluded. 7 drugs (progesterone, rapamycin, everolismus, Deferasirox, auranfin, rimantadine, and amiodarone hydrochloride) which have been considered to be involved in AD pathogenesis were not excluded.


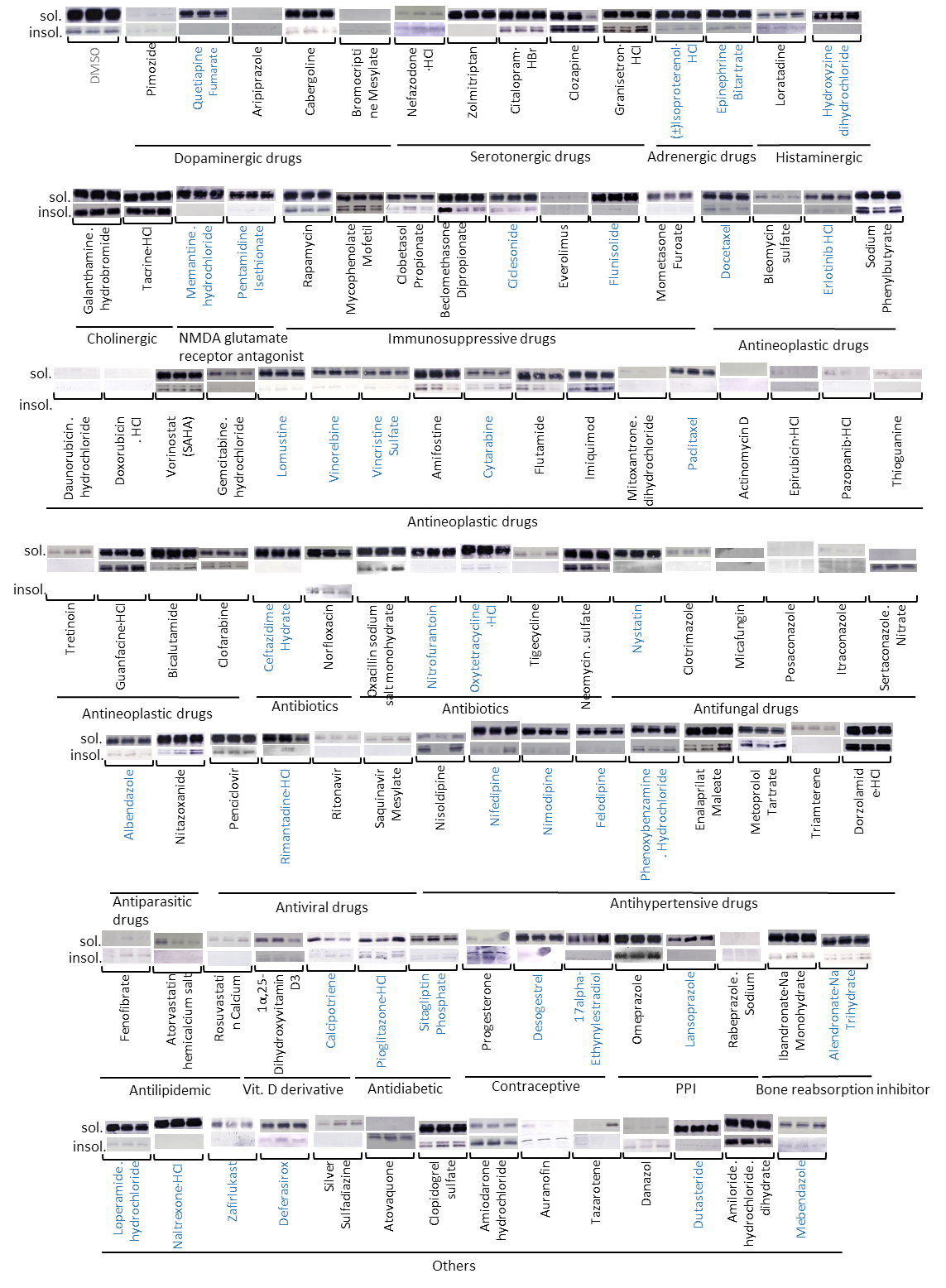


**Supplementary Figure 3.** Analysis of soluble and insoluble tau of 108 drugs at 3rd step. If a compound did not lower the amount of soluble tau but decrease the insoluble tau, it was selected as hit compound in this step. 38 drugs in blue color were selected.


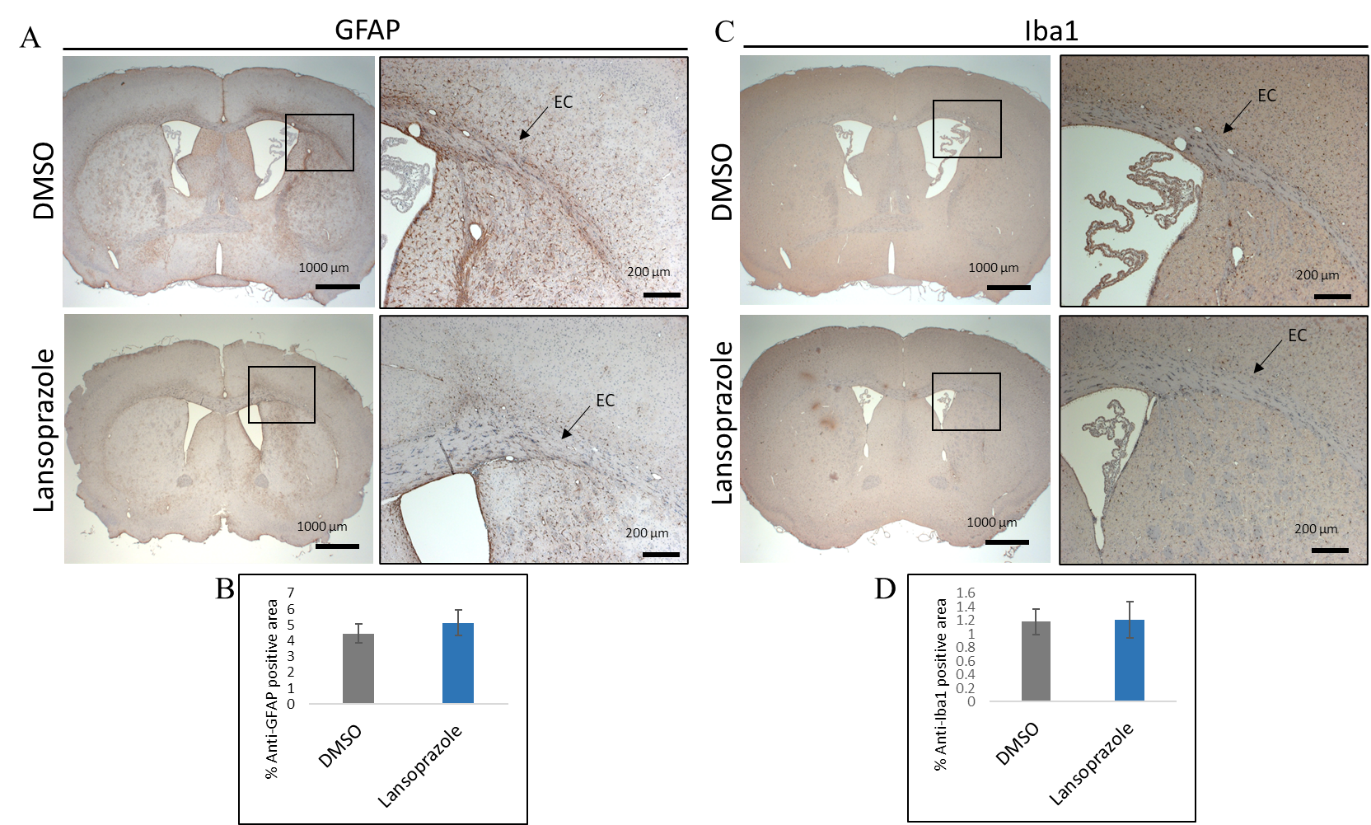


**Supplementary Figure 4.** Lansoprazole treatment did not alter the reaction of brain inflammatory cells. Anti-GFAP antibody immunohistochemistry showed no significant difference between DMSO-treated and lansoprazole-treated mice at the EC and caudate putamen (CPU). (A and B). Anti-Iba1 antibody also did not show any significant difference between DMSO-treated and lansoprazole-treated mice in the same regions (C and D).


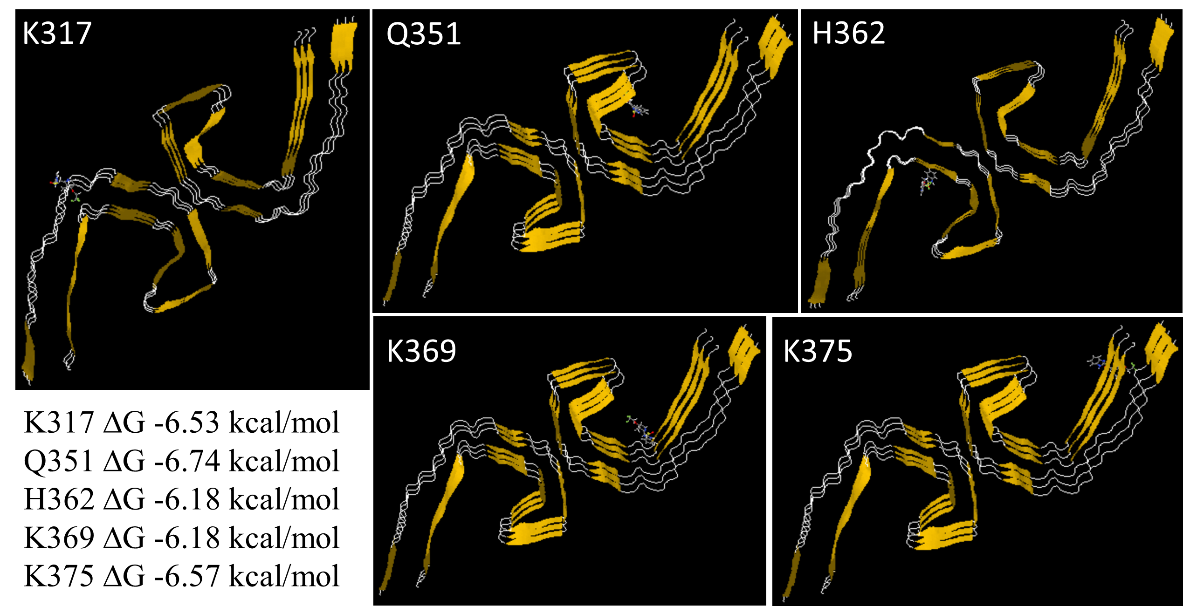


**Supplementary Figure 5.** Predicted binding sites of lansoprazole to paired helical filament from sporadic Alzheimer's disease brain (pdb6HRE) using SwissDock molecular docking prediction tool. Five most stable interaction sites were chosen from 36 predicted sites. Lower ∆G value corresponds to more stable docking.

# Supplementary Tables

**Supplementary Table 1: Description of Alzheimer’s disease cases**

| **Case Name** | **Clinical**  **diagnosis** | **Pathological**  **diagnosis** | **Age at death** | **Duration** | **Postmortem**  **interval** | **Brain region** |
| --- | --- | --- | --- | --- | --- | --- |
| AD 1 | AD | AD  (Braak stage VI) | 84 | 5 | N/A | Frontal Cortex |
| AD 2 | CBS | AD  (Braak stage V) | 73 | 8 | 17 h 43 min | Frontal Cortex |
| AD 3 | AD | AD  (Braak stage VI) | 83 | 13 | 1 h 53 min | Frontal Cortex |

AD: Alzheimer’s Disease; N/A: Not Available; CBS: Corticobasal Syndrome

**Supplementary Table 2: Details of the antibodies**

| **Target** | **Clones** | **Source** | **Application** | **Dilution** | **Detection**  **methods** | **Incubation** |
| --- | --- | --- | --- | --- | --- | --- |
| T46(aa 404-441) | Monoclonal | Invitrogen | Western blotting | 1:1000 | DAB | O/N, RT |
| AT8 | Monoclonal | Invitrogen | IHC | 1:200 | DAB | O/N, 4°C |
| GAPDH | Monoclonal | Millipore | Western blotting | 1:1000 | DAB | O/N, 4°C |

**Supplementary Table 3: Information of primers**

| **Target** | **Template** | **Forward** | **Reverse** |
| --- | --- | --- | --- |
| Lys317 | pCDNA3.1 Tau-CTF24 | 5´-GCCGTGACCTCCAAGTGTGGCTC-3´ | 5´-GCTCAGGTCAACTGGTTTGTAG-3´ |
| Gln351 | pCDNA3.1 Tau-CTF24 | 5´-GCCTCGAAGATTGGGTCCCTGG-3´ | 5´-GACTCTGTCCTTGAAGTCAAGC-3´ |
| His362 | pCDNA3.1 Tau-CTF24 | 5´-GCCGTCCCTGGCGGAGGAAATAAAAAG-3´ | 5´-GGTGATATTGTCCAGGGACCC-3´ |
| Lys369 | pCDNA3.1 Tau-CTF24 | 5´-GCCAAGATTGAAACCCACAAGC-3´ | 5´-ATTTCCTCCGCCAGGGACGTGGG-3´ |
| Lys375 | pCDNA3.1 Tau-CTF24 | 5´-GCCCTGACCTTCCGCGAGAACG-3´ | 5´-GTGGGTTTCAATCTTTTTATTTCC-3´ |

**Supplementary Table 4: Number of drugs selected at 3 steps**

| **Class of drugs** | **1. Number of hit drugs from the first screening** | **2. Number of drugs selected after LDH assay** | **3. Number of drugs selected after soluble and insoluble tau western blot** |
| --- | --- | --- | --- |
| **Neurotransmitter-modulating drugs** |  |  |  |
| **Monoaminerigic drugs** |  |  |  |
| **Dopaminergic drugs** | 6 | 5 | 1 |
| **Serotonergic drugs** | 6 | 5 | 0 |
| **Adrenerigic drugs** | 2 | 2 | 2 |
| **Histaminergic drugs** | 5 | 2 | 1 |
| **Cholinerigic drugs** | 3 | 2 | 0 |
| **NMDA glutamate receptor antagonist** | 2 | 2 | 2 |
| **Immunosuppressive drugs** | 12 | 8 | 2 |
| **Antineoplastic drugs** | 45 | 25 | 7 |
| **Infection-related drugs** |  |  |  |
| **Antibiotics** | 10 | 7 | 3 |
| **Antifungal drugs** | 17 | 5 | 1 |
| **Antiprotozoal drugs** | 3 | 0 | 0 |
| **Antiparasitic drugs** | 2 | 2 | 1 |
| **Antiviral drugs** | 8 | 4 | 1 |
| **Lifestyle disease** |  |  |  |
| **Antihypertensive drugs** | 13 | 9 | 4 |
| **Antilipidemic drugs** | 6 | 3 | 0 |
| **Antidiabetic drugs** | 3 | 2 | 1 |
| **Vitamin D derivates** | 2 | 2 | 2 |
| **Contraceptive drugs** | 4 | 3 | 2 |
| **Proton pump inhibitor** | 3 | 3 | 1 |
| **Bone reabsorption inhibitor** | 2 | 2 | 1 |
| **Others** | 26 | 16 | 6 |
| **Total** | **180** | **108** | **38** |
